# HALT-PROP: Human-Annotated Lithuanian Textual Corpus for Propaganda Narratives and Techniques

**DOI:** 10.1038/s41597-025-06367-w

**Published:** 2025-12-03

**Authors:** Ieva Rizgelienė, Vilma Zubaitienė, Nerijus Maliukevičius, Virginijus Marcinkevičius

**Affiliations:** 1 Vilnius University Institute of Data Science and Digital Technologies, Vilnius, Lithuania; 2 Vilnius University Faculty of Philology Institute of Applied Linguistics, Vilnius, Lithuania; 3 Vilnius University Institute of International Relations and Political Science, Vilnius, Lithuania

**Keywords:** Scientific data, Computer science

## Abstract

In the contemporary technological landscape, propaganda has become one of the most pervasive tools in information warfare. Social media platforms and entire media ecosystems are leveraged to disseminate hostile propaganda aimed at polarizing societies, destabilizing states, and eroding longstanding democratic processes. Malign propaganda is not only common in widely-spoken languages but also targets less-spoken languages to maximize its reach and influence. While progress has been made in developing models capable of detecting propaganda, most advances have focused on high-resource languages. In contrast, low-resource languages continue to face significant limitations, the most critical being the scarcity of annotated datasets. In many regions and countries, such resources are entirely absent. To address this gap, we present the HALT-PROP dataset, the first human-annotated Lithuanian textual propaganda corpus. The corpus comprises two complementary datasets: (1) 2,870 news articles manually labeled by five experts at the article level to identify the presence of propaganda; and (2) a subset of 1,000 articles annotated for specific propaganda techniques and narratives using a cross-annotation approach.

## Background & Summary

With the rapid advancement of artificial intelligence, increasingly sophisticated natural language processing (NLP) methods are being developed to address a wide range of tasks, including the detection of propaganda and disinformation. Due to abundant linguistic data, the main progress has been achieved in rich-resource languages, where machine learning models demonstrate high levels of accuracy in identifying fake news^1^ and propaganda^2^. Progress is also made in low-resource languages^3^, which is mostly related to the development of multilingual transformer-based models^4,5^. These models, pre-trained simultaneously across multiple languages, leverage cross-lingual knowledge transfer to effectively capture both universal and language-specific textual characteristics.

Despite recent advancements, applying machine learning models to propaganda detection in low-resource languages still faces significant limitations. These challenges are common across all NLP tasks for low-resource languages, including limited access to digital data and resources, complex linguistic structures, and a lack of annotated datasets^6^. There remains a significant need for new data resources in low-resource languages. In tasks such as propaganda and disinformation detection, this scarcity is even more pronounced: entire regions and countries often lack any available datasets at all.

### Related work

Although textual corpora specifically tailored for propaganda analysis remain relatively scarce, considerable progress has been made, particularly with English-language resources. The pioneering *TSHP-17* corpus comprises news articles labeled as trusted, satire, hoax, or propaganda, based on the credibility of their originating news outlets^7^. Subsequently, the *Proppy* corpus was introduced, distinguishing between propaganda and trustworthy content^8^. Numerous datasets also explore related aspects of propaganda, including fake news, misinformation, rumors, and veracity. Notable datasets for fake news detection encompass the *LIAR* dataset, which utilizes six distinct truthfulness labels derived from fact-checking sources^9^; *the Political News dataset* with three categories, satire, fake and real news^10^; the comprehensive *FakeNewsNet* repository integrating articles with rich metadata^11^; and the *ISOT* dataset^12^. Additionally, datasets are available for rumor detection and assessing claim veracity, such as *PHEME*^13^, *CREDBANK*^14^, *Emergent*^15^, *FEVER*^16^, and *FNC-1*^17^. All the aforementioned datasets provide labels at the article level, meaning that labels apply to entire texts rather than at more granular levels such as tokens, words, or sentences. Consequently, these datasets do not facilitate an in-depth analysis of specific linguistic aspects, such as the use of propaganda techniques.

In 2020, the SemEval organization, known for its series of international workshops on NLP, introduced propaganda technique detection as Task 11^18^. This task presented the PTC-SemEval20 corpus, an English-language dataset comprising 536 articles collected from 13 propaganda and 36 non-propaganda news media outlets, annotated for 14 distinct propaganda techniques. This corpus was the first human-annotated dataset specifically addressing propaganda techniques at a more granular level, focusing on specific fragments or spans of text rather than labeling entire articles, thus enabling a more detailed and nuanced analysis.

Progress has also been achieved in developing propaganda corpora for languages other than English. SemEval has extended its original 2020 propaganda detection task twice: first in 2023, by introducing the Multilingual Propaganda Detection Dataset, featuring span-level annotations of 23 persuasion techniques, framing annotations, and news genre labels across nine languages (English, French, German, Italian, Polish, Russian, Georgian, Greek, Spanish)^19^; and subsequently in 2024, with a multilingual and multimodal dataset comprising approximately 10,000 English-language memes, supplemented by smaller test sets in Bulgarian, North Macedonian, and Arabic, annotated for 22 persuasion techniques grouped into Ethos, Pathos, and Logos categories^20^.

Regarding pro-Russian propaganda, a comprehensive Czech-language corpus was developed containing 8,646 news articles from four outlets previously identified as distributors of Russian propaganda. This dataset includes fine-grained annotations for manipulative propaganda techniques along with document-level attributes such as genre, sentiment, topic, location and additional textual attributes^3^.

Additional datasets annotated specifically for propaganda techniques include a Russian-language corpus focused on COVID-19 propaganda^21^, an Arabic dataset of 930 tweets annotated for 20 techniques^22^, a multilingual English Roman Urdu dataset comprising 1,030 social media posts annotated for 20 techniques^23^, and a Mandarin corpus consisting of 9,950 annotated tweets for 21 techniques^24^. Furthermore, datasets lacking granular annotations, providing only article-level labels for propaganda, include H-Prop and H-Prop-News for Hindi^25^, as well as various fake news datasets in Spanish^26^, Chinese^27^, and Urdu^28^.

Table 1 summarizes existing propaganda corpora. Since our dataset includes article-level labels to identify the presence of propaganda, as well as span-level annotations for propaganda techniques and labels for expressed narratives, we focused on datasets that contain at least one of these features. Given that our corpus was developed for Lithuanian, a low-resource language, we prioritized corpora created for other low-resource languages. However, due to the limited availability of such resources, we also considered datasets annotated for propaganda features in languages other than English, or in multilingual settings that included English alongside other languages. For completeness, we also include foundational English-language corpora, such as TSHP-17, Proppy, and PTC-SemEval-2020. The columns in Table 1 indicate whether each dataset includes article-level labels, annotations of propaganda techniques, and information related to specific narratives or topics, either as explicit labels or identifiable through annotation features. Additionally, we assessed whether the datasets explicitly address pro-Russian propaganda.

**Table 1:** Publicly available propaganda datasets.

Dataset (Year)	Article-Level Labels	Propaganda Techniques	Narratives or Topics	Pro-Russian Propaganda	Language(s)	Corpus Size
TSHP-17^7^ (2017)	Yes	No	No	No	English	22,580 articles
Proppy^8^ (2019)	Yes	No	No	No	English	51,294 articles
PTC-SemEval20^18^ (2020)	No	Yes	No	No	English	536 articles
Chang *et al*.^24^ (2021)	No	Yes	No	No	Mandarin	9,950 tweets
WANLP^22^ (2022)	No	Yes	No	No	Arabic	930 tweets
H-Prop^25^ (2022)	Yes	No	No	No	Hindi	28,630 articles
H-Prop-News^25^ (2022)	Yes	No	No	No	Hindi	5,500 articles
SemEval-2023 Task 3 Dataset^19^ (2023)	No	Yes	No	No	English, French, German, Italian, Polish, Russian	2,000 articles Mbzuai-nlp
/propaganda-codeswitched-text^23^ (2023)	No	Yes	No	No	English, Roman Urdu	1,030 Social media posts
ZenPropaganda^21^ (2024)	No	Yes	No	No	Russian	125 texts
MU-NLPC/Propaganda^3^ (2024)	No	Yes	Yes	Yes	Czech	8,646 articles
SemEval-2024 Task 4^20^ (2024)	No	Yes	No	No	English, Bulgarian, North Macedonian, Arabic	10,000 memes

The analysis reveals that while several corpora exist for low-resource languages annotated for propaganda techniques, none are dedicated specifically to the languages of the Baltic countries, or even to those of Russia’s neighboring states, regions frequently targeted by Russian propaganda. Furthermore, we found that only one existing corpus explicitly incorporates propaganda narratives or topics, despite their central role in the dissemination of propaganda messages. Regarding pro-Russian propaganda, only a single dataset, focused on the Czech language, directly addresses this issue. These findings highlight the uniqueness and significance of our Lithuanian propaganda corpus. It is the first annotated dataset developed for Lithuanian, and, more broadly, for any Baltic or Russia-bordering language. Uniquely, it includes both propaganda technique annotations and explicit labels for propaganda narratives, addressing a critical but largely neglected dimension in existing resources.

### Motivation

Despite notable progress in developing propaganda-related textual corpora for low-resource languages, resources specifically targeting Russian propaganda in the native languages of Russia’s immediate neighbors and other post-communist states remain limited. For low-resource languages in this region, textual datasets focused explicitly on Russian propaganda are scarce, with the notable exception of a comprehensive Czech-language corpus^3^. It is also important to note that no dedicated propaganda corpora currently exist for the languages of the Baltic countries (Lithuanian, Latvian, Estonian), nor for other languages spoken in Russia’s neighboring states, such as Ukrainian, Kazakh, Azerbaijani, or Georgian.

This gap is especially striking, given that Russian information operations have long prioritized targeting the so-called “near abroad”^29^. Numerous studies have highlighted this pattern^30^. This Russian approach is rooted in doctrines such as the “humanitarian dimension of foreign policy”^31^, which frame the neighboring countries not only as spheres of privileged interest but also as ideological and cultural extensions of Russia. Although substantial research has examined the Russian malign influence toolbox, focusing on actors, platforms, and tactics^32–34^, few efforts have been sought to systematically document or annotate propaganda texts in the native languages of the targeted societies. This lack of native-language corpora hinders the development of AI-driven detection tools, automated narrative analysis, and multilingual disinformation monitoring systems. This is particularly problematic in the Baltic region, where Lithuania, Latvia, and Estonia remain on the frontlines of Russian information warfare, but lack dedicated linguistic resources for the computational analysis of propaganda content. Addressing this gap is critical for advancing both academic understanding and practical measures against Russian malign influence in vulnerable language ecosystems.

## Methods

The aim in creating this dataset was to create a dataset that is suitable to train/fine-tune large language models for propaganda identification in media outlets. To successfully achieve this aim, we did a deep analysis in order to correctly define and describe what propaganda is, what propaganda techniques are, and what topics are most sensitive and popular for propaganda in a information war context.

### Preparation process

In this preparation process, we define propaganda, propaganda narratives and techniques. Later, these definitions were used by annotators to uniquely identify them.

### Definition of propaganda

Propaganda can be defined in many ways: it can be described as ideas or statements that may be false or present only one side of an argument that is used to gain support for a political leader, party, etc^35^, a systematic effort to shape perceptions and behavior^36^, messaging that exploits emotional rather than rational responses^37^, or methods employed by organized groups to psychologically unify and mobilize individuals^38^. Broadly, propaganda involves deliberate communication strategies designed to influence public opinion through specific topics, narratives, and techniques.

In propaganda analysis, a topic denotes the core issue or theme being discussed, while a narrative refers to the specific framing or interpretation applied to that topic, combining facts, values, and emotions into a coherent message. In our study, focused on pro-Russian propaganda aimed at polarizing and radicalizing Lithuanian society, we classify an article as propagandistic if it explicitly presents at least one of our identified propaganda narratives, and employs at least one recognized propaganda technique.

### Classification and explanation of propaganda techniques

This classification groups related propaganda techniques into broader categories based on shared rhetorical strategies and persuasive functions, reflecting their conceptual similarities. Building on SemEval datasets, which show that grouping similar techniques improves annotation accuracy and clarity, this framework simplifies the approach by consolidating various emotional appeals under “Emotional Expression” and merging “Doubt” with “Smears.” Clear definitions accompany each technique to support conceptual clarity and interdisciplinary use. Prioritizing usability over fine-grained differentiation, it identifies ten distinct techniques, mainly for qualitative and interdisciplinary analysis, rather than automated processing.

In contrast, the SemEval-2023 Task 3 framework by Piskorski *et al*.^19^ offers a more detailed scheme with 23 techniques, distinguishing closely related strategies such as “Whataboutism,” “Red Herring,” and “Straw Man” separately, and splitting emotional appeals into “Loaded Language,” “Appeal to Fear-Prejudice,” and “Exaggeration-Minimisation.” Some categories in the original classification, like “Reductio ad Hitlerum/Stalinum,” are absent from SemEval-2023, which includes other categories merged or missing in the original. These differences reflect contrasting priorities: simplicity and broad applicability for qualitative analysis, versus detailed differentiation for computational annotation in multilingual contexts.

To maintain clarity, this simplified classification integrates SemEval-exclusive techniques into broader categories: Slogans fall under Simplification as catchy phrases simplifying complex issues; Conversation Killer aligns with Doubt/Smears or Intentional Vagueness due to its role in disrupting discussion and evading scrutiny; Obfuscation-Vagueness-Confusion is grouped under Intentional Vagueness; Loaded Language and Exaggeration-Minimisation belong to Emotional Expression, with the latter also fitting under Simplification for selective emphasis.

The SemEval-2024 techniques “Thought-Terminating Cliché,” “Glittering Generalities,” and “Presenting Irrelevant Data” are incorporated as follows: “Thought-Terminating Cliché” fits under Simplification for shutting down critical thinking; “Glittering Generalities” is part of Emotional Expression due to its vague, positive language evoking emotion; and “Presenting Irrelevant Data” aligns with Doubt/Smears or Intentional Vagueness, as it distracts from the main issue^20^. This grouping simplifies detailed distinctions for clearer qualitative analysis.

By situating these techniques within broader categories, the classification balances accessibility and usability for qualitative and interdisciplinary analysis, while acknowledging SemEval’s emphasis on fine-grained distinctions. The table below presents the ten propaganda techniques identified here, with explanations to support clear understanding and application.

Alongside defining and categorizing propaganda techniques, it is equally important to examine how these techniques manifest within specific narratives.

### Selection of propaganda narratives

The next stage involved identifying specific Russian-originated narratives directed at Lithuania. Drawing on existing research and institutional assessments, a list of key propaganda narratives attributed to Russia was compiled. This list served as the basis for the annotation process and enabled systematic analysis and categorization of propaganda narratives in the dataset.

While the form of storytelling, how messages are structured and delivered, is the focus of propaganda technique analysis, this study adopted Catherine Kohler Riessman’s thematic narrative analysis to examine the content of propaganda messaging. This dual-level approach allows distinguishing between the deeper narrative structures and the rhetorical techniques used to reinforce them. Thematic analysis, as defined by Riessman, emphasizes “what” is said rather than “how” it is said, aiming to identify recurring conceptual groupings and thematic units across cases^39^. Propaganda narratives can be analyzed not merely as collections of false claims, but as coherent meaning-making structures that organize information into politically consequential storylines. Patterson and Monroe describe narrative as “the ways in which we construct disparate facts in our own worlds and weave them together cognitively in order to make sense of our reality”^40^. Accordingly, identifying Russian propaganda narratives against Lithuania involves tracing how malign actors selectively assemble events, symbols, and moral judgments into strategic narratives that seek to normalize anti-Lithuanian worldviews.

Accordingly, existing research on Russian propaganda in Lithuania and the broader region was reviewed in order to extract already-identified narrative themes. These served as the basis for a systematic annotation scheme that enabled the classification of messages into recurring ideological and thematic categories. The 2018 report by NATO StratCom COE “Russia’s Footprint in the Nordic-Baltic Information Environment” identified a total of 29 narratives promoted by Russia in regard to the Nordic-Baltic countries^41^. The 2020 follow-up report “Information Influence Campaigns in the Baltic States” further refined the typology, e.g. some narratives were added, and some were removed from the list^42^. Among the Russian propaganda narratives targeting the wider Baltic-Nordic region, several were specifically adapted to the Lithuanian context. These could be summed up as following:

- NATO as a threat: portraying NATO’s presence in Lithuania as provocative and destabilizing.
- Historical revisionism: denying the Soviet occupation of Lithuania, questioning the legitimacy of Lithuanian independence.
- Failed state: depicting Lithuania as a failed and irrelevant state, manipulated by foreign powers.
- Russophobia: alleging systemic discrimination against Russian-speakers.
- Western moral decay: contrasting Western immorality with Russian traditional values.
- Geopolitical victimhood: presenting Russia as encircled by enemies (Lithuania, NATO), shifting the blame for regional tensions away from Russian actions.
- Migration and refugees: framing Lithuania’s policy in this sector as a threat to national identity, security, and social cohesion.
- Questioning rearmament and defence spending: criticising Lithuania’s increased defence budget and military modernisation as unnecessary or externally imposed.
- The Baltics as geopolitical playground: portraying Lithuania as a puppet of global political-economic elites, particularly George Soros and the Washington establishment.

In addition to the propaganda narratives already identified in prior research, it was essential to incorporate the assessments provided by Lithuanian state institutions. Equally important was the need to contextualize the observed narratives within the broader political and social environment, namely, the key events that occurred during the period covered by the annotated corpus. As a first step, the annual National Threat Assessments, published by Lithuania’s State Security Department and the Defence Intelligence and Security Service, were analyzed. These reports offer a perspective on how state national security institutions evaluate Russian propaganda narratives targeting Lithuania. The 2023 report outlined two key narratives that focused on the war in Ukraine: the first one targeted Western sanctions against Russia; the second Ukrainian refugees^43^. The 2024 report added three more anti-Ukraine related narratives: war in the Baltic states is imminent; support for Ukraine is pointless; the war in Ukraine is of no importance to the West^44^. Building on the previous analysis, the 2025 report further underscored that, alongside anti-Ukrainian propaganda, Russia had revived its traditional narratives directed against Lithuania: first, Russia sought to discredit Lithuania by accusing it of rewriting history, promoting Nazism, and fostering Russophobia, framing Lithuania as one of the most aggressively anti-Russian countries in Europe; second, historical revisionist narratives accused the Baltic states of distorting the history of the Second World War, glorifying Nazi collaborators, and erasing Soviet contributions; the third narrative claimed systematic discrimination against Russian speakers in Lithuania, aiming to attract criticism from international human rights institutions, and to equate Lithuania’s domestic policies with historical atrocities^45^. Moreover, when assessing the broader geopolitical disruptions that shaped the information environment during this period, developments in Belarus must be considered. These include the refugee crisis at the Lithuanian border, orchestrated by the Lukashenko regime, and the mass protests following the fraudulent presidential elections. The migration wave was accompanied by coordinated propaganda campaigns, while the subsequent flight of Belarusians from the Lukashenko regime created new societal tensions in Lithuania. Russian and Belarusian propaganda actors have exploited these developments, particularly by reviving historical themes related to “Litvinism”, to sow mistrust in Lithuania towards Belarusians^46^.

These narrative themes provided an academically and contextually grounded foundation for the annotation process and were synthesized for the empirical coding phase as follows, to reflect both continuity and evolution in hostile messaging patterns:

1. Disinformation about the war in Ukraine (i.e. spreading false narratives to justify Russia’s aggression and delegitimize Ukrainian resistance);
2. Delegitimization of the Lithuanian State (i.e. slandering the Republic of Lithuania as a failed or artificial “project,” questioning its sovereignty and historical foundations);
3. Undermining the Lithuanian Armed Forces (i.e. attacks on military funding, modernization efforts, and NATO deployments, aiming to portray Lithuania as militaristic or provocatively anti-Russian);
4. Erosion of Trust in Lithuanian Institutions (i.e. promoting narratives that depict state authorities as corrupt, incompetent, or unrepresentative);
5. Attacks on Western Institutions and Alliances (i.e. discrediting the EU, NATO, and other multilateral bodies, framing them as exploitative, ineffective, or morally bankrupt);
6. Decline of Western Civilization (i.e. spreading claims about Western moral decay, often emphasizing themes like gender ideology, LGBT rights, or secularism, to contrast it with “traditional values”);
7. Authoritarian Model Promotion (i.e. highlighting regimes like Moscow, Minsk, or Beijing as examples of stability, efficiency, and sovereign governance in contrast to Western “chaos”);
8. Narratives of US Decline and “Washington Hegemony” (i.e. framing the United States as a waning imperial power and suggesting the emergence of a multipolar or “New World Order” led by Russia and China);
9. Geopolitical Reordering and the “New World Order” (i.e. promoting conspiracy-laden ideas about a global realignment that replaces liberal democratic systems with alternative authoritarian alliances);
10. Weaponization of Migration and Refugees (i.e. amplifying fears around migrant flows and depicting them as tools of hybrid warfare or existential threats to national identity and security);
11. Revival of “Litvinism” (i.e. exploiting historical revisionism to push the narrative that parts of Lithuania historically belonged to Belarus, undermining the Lithuanian national identity and territorial integrity).

### Data collection process

In the initial stage of selecting media outlets for articles for the dataset, we referenced findings from research^47^ conducted by Lithuanian National Radio and Television (LRT) and the independent disinformation analysis centre DebunkEU.org, which provided a detailed analysis of Lithuanian online networks disseminating pro-Kremlin disinformation, particularly during the initial phase of Russia’s invasion of Ukraine. Starting with a list of potential sources for the dataset, we analyzed these media outlets to assess their current accessibility, as several were shut down during 2024. We also investigated the presence and activity of these media outlets on social media to identify the most influential ones. During this investigation, we found that some sources are just republishing information from other sources. Therefore, they were excluded from the candidate list. All sources that were ranked and chosen for our dataset were identified by this investigation as key distributors of misleading narratives, conspiracy theories, and propaganda intended to distort public perceptions, legitimise Russian military actions, and undermine trust in Western democratic institutions. In Table 3, we present all media outlets chosen as the text basis for the dataset. The texts were collected during the period from 1 January 2018 to 1 October 2024. This period was chosen to see how propaganda techniques and narratives changed before and during the war. Not all media outlets’ texts could consist of propaganda narratives. Therefore, we chose specific categories (topics) for the data collection. For future analysis, we also chose one media outlet (Lrt.lt) that is sponsored by the Lithuanian government; therefore, we assume it should not have any propaganda texts, but could be very valuable to validate our annotation results. For the same reason, we also did not remove news articles based on topics (e.g. sport, leisure).

**Table 2:** Classification and explanation of common propaganda techniques.

No.	Technique	Explanation
**1**	**Emotional Expression**	Intentionally uses emotionally charged language (e.g., fear, anger, pride, sympathy) to provoke strong feelings and influence audience beliefs or actions. Often avoids logical reasoning and relies on exaggeration, personal attacks, or vague but positive terms to shape perception.
**2**	**Whataboutism/ Red Herring/Straw Man**	Distracts from the main issue by shifting blame or criticism to others (Whataboutism), introducing irrelevant information or arguments (Red Herring), or misrepresenting an opponent’s view by exaggerating, distorting, or oversimplifying it to attack a weaker version (Straw Man). These strategies serve to divert or deflect attention from the core argument.
**3**	**Simplification**	Deliberately reduces complex issues to overly simple explanations by attributing blame or responsibility to a single cause or group, framing problems as having only two opposing options, using stereotyped phrases that shut down deeper thinking, and relying on short, catchy slogans that appeal more to emotions than logic. These strategies limit critical analysis and obscure the true complexity of issues.
**4**	**Intentional Vagueness (Obfuscation)**	Uses ambiguous or imprecise language to obscure meaning, allowing multiple interpretations and helping avoid accountability or direct scrutiny.
**5**	**Appeal to Authority**	Refers to perceived authoritative figures or institutions to legitimize a claim, implying it is true based solely on the authority’s status, often without supporting evidence.
**6**	**Flag-Waving**	Promotes a position by invoking patriotism or national pride, suggesting the idea serves the country’s interests, even in the absence of a clear rationale or evidence.
**7**	**Bandwagon**	Encourages alignment with a belief or action by implying widespread acceptance. Leverages social pressure and the desire to conform to persuade individuals to adopt the majority view.
**8**	**Doubt/Smears**	Seeks to undermine credibility by casting suspicion or attacking character—either subtly (Doubt) or directly through baseless accusations or insinuations (Smears)—without presenting concrete evidence.
**9**	**Reduction ad Hitlerum/Stalinum**	Discredits a person, idea, or group by associating them with historically vilified figures (e.g., Hitler, Stalin), appealing to emotion rather than addressing the actual argument.
**10**	**Repetition**	Reinforces a message through frequent repetition. Over time, repeated statements may appear more familiar and thus more believable, even in the absence of evidence—a psychological effect known as the “illusion of truth.”

**Table 3:** Media outlets chosen for the dataset formation.

Source/Media outlet	Number of topics	Number of items collected	Number of items after the filtration
Būkimevieningi.lt	50	7,409	6,787
Ekspertai.eu	132	13,052	10,367
Infa.lt	82	6,996	6,491
Komentaras.lt	68	1,893	1,403
Ldiena.lt (ldiena.com)	50	18,416	15,583
Lrt.lt	54	139,845	133,197
Minfo.lt	94	15,880	15,530
Total:	203,491	186,376	

At the next stage, the collected data was filtered to remove articles that have the same name or the same content, or have a content length shorter than 200 characters or longer than 10,000 characters. Short texts do not contain any information or enough information to identify them as propaganda or not. On the other hand, the very long pages are hard to analyse and annotate.

### Data selection process

**Fig. 1:**
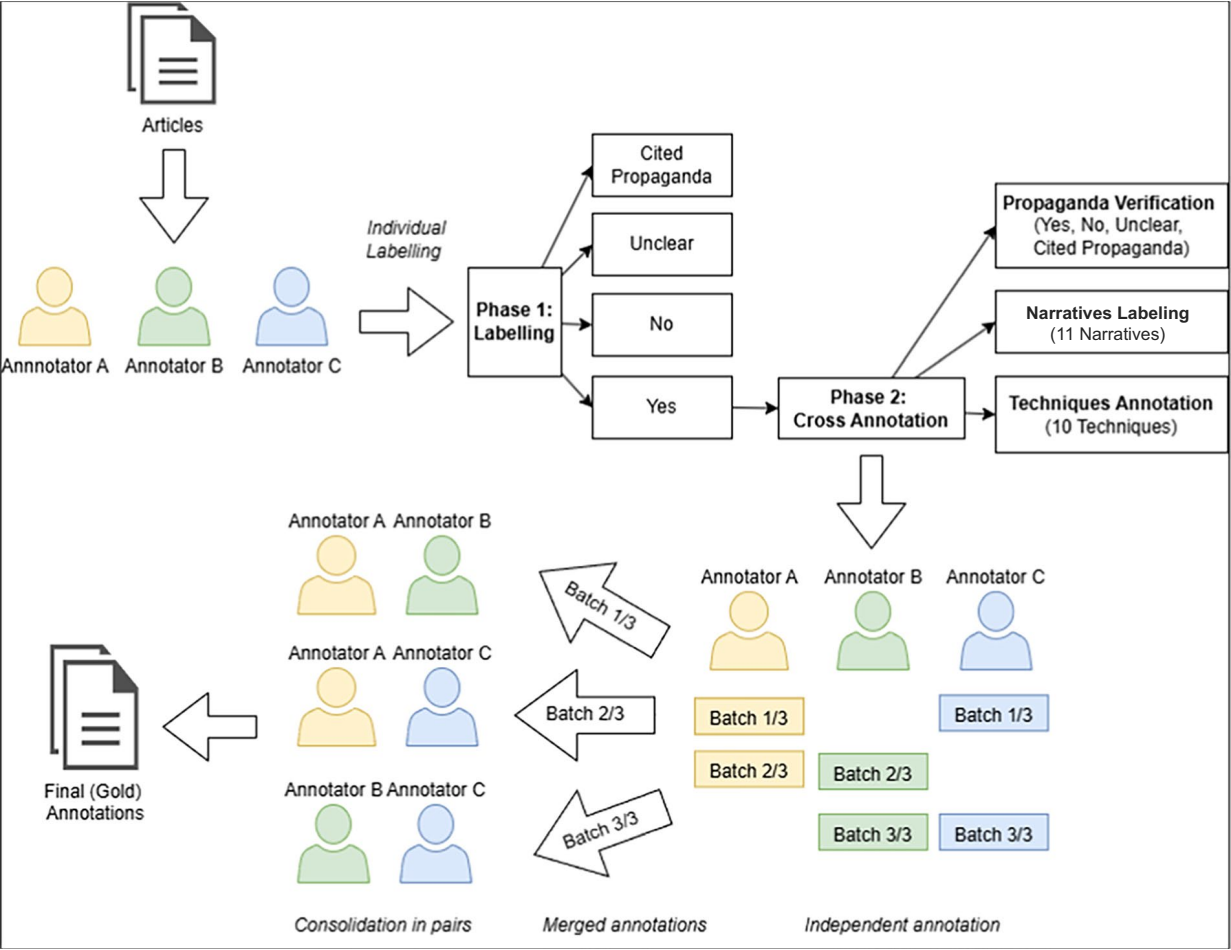
The annotation process.

Items from the collected dataset (see Table 3) were selected randomly in batches of 50 to 100 articles for each media outlet individually for the first stage of the annotation process (see Fig. 1). There were no other criteria to select articles for the annotation.

### Annotation process

Since our goal was to create a propaganda corpus, we needed to annotate articles not only for specific techniques and narratives, but also to identify and filter out propagandistic content. Therefore, our collected articles underwent a two-phase annotation process, as illustrated in Fig. 1. Five annotators were involved in this annotation process. Annotator 1 participated throughout the entire annotation period, while Annotators 2 and 3 contributed during the earlier stages and were later replaced by Annotators 4 and 5 in the later stages. In total, six distinct annotator pairs were formed: three pairs (comprising Annotators 1, 2, and 3) were active in the earlier phase, and three different pairs (comprising Annotators 1, 4, and 5) annotated the corpus during the later phase.

In the first phase, annotators individually assessed whether articles contained propagandistic content by assigning each article one of four possible labels: “yes,” “no,” “propaganda citation,” or “unclear.” An article was labelled “propaganda” (“yes”) if it contained at least one identified propaganda narrative and employed at least one propaganda technique within the text. An article was labelled “not propaganda” (“no”) if neither a propaganda narrative nor a propaganda technique was identified. Two additional labels were also available: “unclear” and “propaganda citation.” The “unclear” label was assigned if an article contained only propaganda techniques without an identifiable propaganda narrative, or, vice versa, only a propaganda narrative without the presence of any propaganda techniques. The “propaganda citation” label was used when propagandistic statements were directly quoted or cited from other sources. This initial phase primarily served to select articles suitable for subsequent detailed annotation. During this phase, each annotator individually reviewed and labelled approximately 50 articles per week, resulting in around 150 articles annotated weekly by the three annotators. The main objective was to identify articles suitable for detailed cross-annotation, aiming for at least 30 articles positively identified as propagandistic (“yes”) per week. If fewer than 30 articles were labelled “yes” from the initial batch of 150, annotators continued reviewing additional articles until the threshold of 30 “yes” articles was achieved.

In the second phase, each week a batch of 30 articles identified as propagandistic in the first phase was selected for propaganda techniques and narratives annotation. These 30 articles were distributed among the annotators so that each annotator was assigned 20 articles. Each article was annotated independently by two different annotators (ten articles overlapping between annotators). After individual annotations were completed, the annotations for each article were merged, and pairs of annotators held weekly discussions to resolve any conflicts, thereby preparing a finalized, consensus-based annotation for each article.

**Fig. 2:**
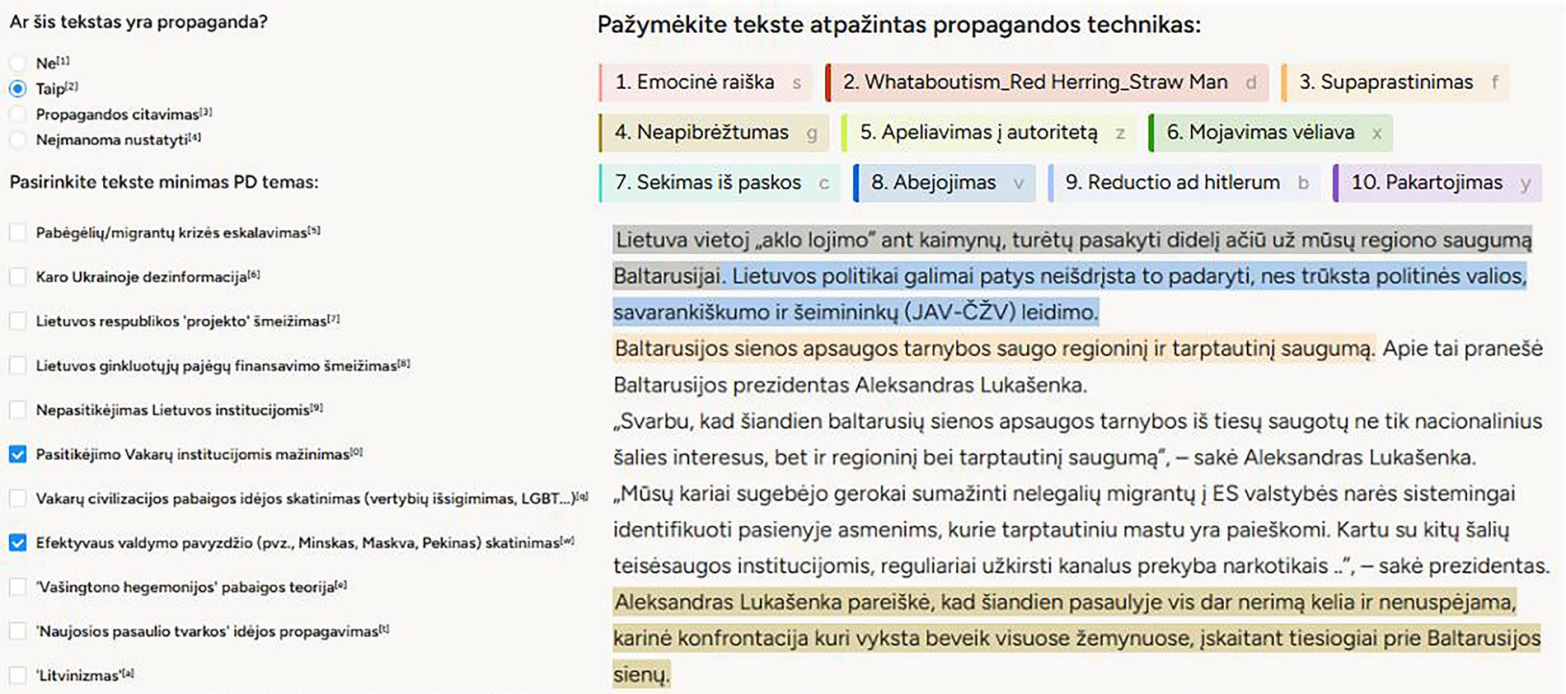
Gold-standard span- and article-level annotation for a single example in Label Studio.

The entire annotation process was conducted in Label Studio, an open-source data labeling platform. We used its built-in tools for span annotation and article-level labeling, and adapted the interface to align with our annotation methodology. Figure 2 presents a gold-standard annotation example for a single article in Label Studio.

### Cross-annotation methodology

As one of the primary goals in developing our propaganda corpus was to minimise the risk of annotator bias, for the second phase of the annotation, a cross-annotation method was selected, as this approach ensures that each document is annotated independently by multiple annotators^48^. As was previously mentioned, five different annotators participated in the annotation process, from which six distinct annotator pairs were formed. Below, we provide the formal definition of the cross-annotation process used in our study.

Let $A=\{{a}_{1},{a}_{2},{a}_{3},{a}_{4},{a}_{5}\}$ denote the set of five annotators involved in the annotation task. The annotators were systematically grouped into six distinct annotation pairs, defined as follows:

$$P=\{\left({a}_{1},{a}_{2}\right),\left({a}_{1},{a}_{3}\right),\left({a}_{2},{a}_{3}\right),\left({a}_{1},{a}_{4}\right),\left({a}_{1},{a}_{5}\right),\left({a}_{5},{a}_{6}\right)\}$$

Each pair ${p}_{i}\in \{1,\ldots ,6\}$ annotated a subset of articles. The annotation procedure consisted of two distinct stages:

- **Stage 1** *(Independent annotation*): Each annotator within pair ${p}_{i}=({a}_{j},{a}_{k})$ independently annotated the assigned articles, producing individual annotation sets ${U}_{j}$ and ${U}_{k}$, respectively.

- **Stage 2** (*Consolidation*): Annotators ${a}_{j}$ and ${a}_{k}$ within each pair ${p}_{i}$ convened to reconcile differences from their independent annotations. This process resulted in a final consolidated annotation (golden standard) ${c}_{i}$, where ${c}_{i}$ corresponds uniquely to a pair ${p}_{i}$.

Thus, for each annotation pair ${p}_{i}\in P$, the final consolidated annotation (gold annotation) is denoted by: ${c}_{i}$ for $i\in \{\mathrm{1,2,3,4,5,6}\}$, where each ${c}_{i}$ serves as the reference annotation for evaluating the annotations of annotators ${a}_{j}$ and ${a}_{k}$ within the respective pair ${p}_{i}$.

**Fig. 3:**
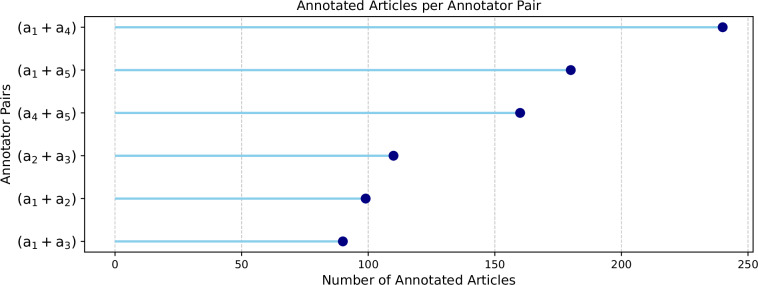
Annotations distribution among the pairs.

Only golden annotations were included in the final propaganda corpus, and, as previously mentioned, these golden annotations were derived from the annotations of two annotators. Figure 3 illustrates the distribution of annotated articles among all annotator pairs.

## Data Records

The HALP-PROP corpus 53 is divided into two files:

- Annotations.csv has information about the article and deep annotation information (whether it is propaganda, narratives, techniques) (see details in Table 7)
- Primary_filter.csv contains information about article (heading, content) and an answer to the question “is it propaganda?” (see details in Table 7)

Both CSV files have UTF-8 encoding and semicolon (“;”) delimiters for column separation. For convenience, we included an XLSX version of the files, too.

## Technical Validation

### Validation of the propaganda technique annotations

To evaluate the quality of the propaganda technique annotations, the γ inter-annotator agreement metric^49^ was employed. This metric is designed for tasks where both spans and their labels, in our case, propaganda techniques, must be identified. Moreover, it can effectively handle overlaps in annotations, which frequently occur in propaganda technique annotation, as multiple different techniques may be assigned to the same textual unit (token, word, sentence, etc). Below, we briefly introduce the methodology and mathematical definitions underlying the γ metric, which was selected to validate the quality of the propaganda technique annotations in our newly created corpus.

#### Definition of annotation units

An annotation unit $u$ identified by annotator $a$ can be defined as: $u=(\left[{u}_{{start}},{u}_{{end}}\right],{u}_{{label}})$, where ${u}_{{start}}$ and ${u}_{{end}}$ denote the start and end character offsets of the annotated span within the text, respectively, and ${u}_{{label}}$ denotes the specific propaganda technique label assigned to this span.

#### Calculation of observed disagreement ($${D}_{o})$$

Observed disagreement measures how different the annotations provided by annotators are. It is computed by aligning annotation units from two annotators to minimize the total disagreement:

- **Spans-only disagreement (**${{\boldsymbol{D}}}_{{\boldsymbol{o}}}^{{\boldsymbol{s}}}$**):** Positional discrepancies between two spans ${u}^{j}$ (annotator ${a}_{j}$) and ${u}^{k}$ (annotator ${a}_{k}$) were calculated as follows:
  $${d}_{{pos}}\left({u}^{j},{u}^{k}\right)={\left(\frac{\left|{u}_{{start}}^{j}-{u}_{{start}}^{k}\right|+\left|{u}_{{end}}^{j}-{u}_{{end}}^{k}\right|}{\left({u}_{{end}}^{j}-{u}_{{start}}^{j}\right)+\left({u}_{{end}}^{k}-{u}_{{start}}^{k}\right)}\right)}^{2}$$
  Given two annotators ${a}_{j}$ and ${a}_{k}$, with aligned annotations, the observed disagreement for spans-only is:
  $${D}_{o}^{s}=\frac{1}{|U|}\sum _{({u}^{j},{u}^{k})\in {alignemnt}}{d}_{{pos}}({u}^{j},{u}^{k}),$$
  where $|{U}|$ represents the average number of annotated units per annotator, and alignment refers to the optimal pairing of annotation units from annotators ${a}_{j}$ and ${a}_{k}$.
- **Span and label agreement (**${{\boldsymbol{D}}}_{{\boldsymbol{o}}}^{{\boldsymbol{sl}}}$**):** combines positional discrepancies with a categorical label difference, calculated by the combined dissimilarity ${d}_{{comb}}$:

$${d}_{{comb}}\left({u}^{j},{u}^{k}\right)=\alpha \cdot {d}_{{pos}}\left({u}^{j},{u}^{k}\right)+\beta \cdot {d}_{{cat}}({u}_{{label}}^{j},{u}_{{label}}^{k}),$$

with weights set as $\alpha =1$ (for positional discrepancies) and $\beta =2$ (for categorical discrepancies), where categorical dissimilarity is defined as:

$${d}_{{cat}}\left({u}_{{label}}^{j},{u}_{{label}}^{k}\right)=\left\{\begin{array}{c}0,{if}\;{u}_{{label}}^{j}={u}_{{label}}^{k}\\ 1,{otherwise}\end{array}.\right.$$

Thus, the observed disagreement combining spans and labels is:

$${D}_{o}^{{sl}}=\frac{1}{|U|}\sum _{({u}^{j},{u}^{k})\in {alignemnt}}{d}_{{comb}}({u}^{j},{u}^{k})$$

#### Calculation of expected disagreement (*D*_*e*_$$)$$

Expected disagreement ${(D}_{e})$ represents the disagreement that would occur by random chance. It is estimated by generating random annotations (with the same distribution of span lengths and counts as observed annotations) and averaging their disagreement. Expected disagreement for span-only case ${(D}_{e}^{s})$ and for span-and-label case ${(D}_{e}^{{sl}})$ were computed by simulation, using random annotation samples ${D}_{e}=E[{D}_{o}^{{random}}]$.

#### Final Gamma Calculation (γ)

The final gamma (γ) inter-annotator agreement measure is computed as the normalized difference between observed and expected aggreements:

- **Span-only gamma (**${{\boldsymbol{\gamma }}}_{{\boldsymbol{s}}}$**):**
  $${\gamma }_{s}=1-\frac{{D}_{o}^{s}}{{D}_{e}^{s}}.$$
- **Span-and-label gamma (**${{\boldsymbol{\gamma }}}_{{\boldsymbol{sl}}}$**):**

$${\gamma }_{{sl}}=1-\frac{{D}_{o}^{{sl}}}{{D}_{e}^{{sl}}}.$$

### Inter-annotator agreement analysis results

Table 4 presents the results of the gamma (γ) inter-annotator agreement analysis. Span-only agreement between annotators ranged predominantly from 0.10 to 0.36, suggesting considerable disagreement regarding the exact textual boundaries of annotated propaganda instances. When considering both spans and labels, agreement further dropped to a range of 0.07 to 0.25, revealing substantial additional discrepancies in labeling propaganda techniques. However, when comparing individual annotators’ results against consolidated gold annotations, agreement notably improved. Span-only agreement with gold annotations ranged from 0.31 to 0.69, demonstrating that consolidation significantly enhanced clarity in identifying spans. Agreement on both spans and labels with gold annotations ranged from 0.43 to 0.76. Considering that the annotations were produced entirely by human annotators, some variability and discrepancies were expected. Similar patterns and agreement levels were observed in the SemEval propaganda techniques corpus^50^, where initial inter-annotator agreement values for spans ranged from 0.30 to 0.34, and for spans and labels from 0.24 to 0.28. Their consolidated agreement with gold annotations ranged from 0.42 to 0.76 for spans only, and from 0.39 to 0.74 when labels were considered as well. The results from our corpus are therefore comparable to the SemEval corpus. Although our initial annotator agreement values exhibit a wider range, this is primarily due to the greater complexity of our annotation setup: five annotators formed six different annotation pairs in our study, whereas the SemEval corpus involved four annotators organized into only two pairs. This more extensive pairing structure in our study contributes to increased diversity in annotations, and further reduces potential annotator bias.

**Table 4:** Gamma (γ) inter-annotator agreement between annotators spotting spans alone (spans) and spotting spans + labelling (+labels).

*Agreement between annotators*			*Agreement between the annotator and the final golden annotation*			
Annotations of the annotator pair	Spans (*γ*_*s*_)	+labels (γ_sl_)	Annotations		Spans (*γ*_s_)	+labels (γ_sl_)
($${a}_{1}+{a}_{2})$$	0.32	0.22	$${a}_{1}$$	$${c}_{1}$$	0.52	0.56
			${a}_{2}$	${c}_{1}$	0.59	0.69
$({a}_{1}+{a}_{3})$	0.1	0.07	${a}_{1}$	${c}_{2}$	0.61	0.67
			${a}_{3}$	${c}_{2}$	0.31	0.43
$({a}_{2}+{a}_{3})$	0.23	0.19	${a}_{2}$	${c}_{3}$	0.69	0.76
			${a}_{3}$	${c}_{3}$	0.35	0.46
($${a}_{1}+{a}_{4})$$	0.35	0.25	$${a}_{1}$$	$${c}_{4}$$	0.64	0.68
			${a}_{4}$	${c}_{4}$	0.54	0.6
$\left({a}_{1}+{a}_{5}\right)$	0.29	0.16	${a}_{1}$	${c}_{5}$	0.67	0.71
			${a}_{5}$	${c}_{5}$	0.45	0.53
($${a}_{4}+{a}_{5})$$	0.36	0.25	$${a}_{4}$$	$${c}_{6}$$	0.64	0.71
			${a}_{5}$	${c}_{6}$	0.48	0.55

Table 5 presents γ-inter annotator agreement results for each propaganda technique. The highest agreement occurred for “repetition,” “following behind,” and “waving the flag,” as these techniques have clear indicators, like repeated phrases, encouragement of group conformity, and patriotic appeals. The combined technique Whataboutism/Red Herring/Straw Man yielded the lowest inter-annotator agreement, reflecting the difficulty of consistently identifying topic diversion or misrepresentation. It was relatively rare in our corpus (3.74% of spans), so even small inconsistencies could noticeably lower agreement. Moreover, this challenge is not unique to our study: in SemEval-2020 Task 11, participating teams reported among the lowest classification scores for this technique, indicating broader difficulties for automated systems^51^. Moderate agreement was observed for “appeal to authority” and “reductio ad Hitlerum.” Although these techniques involve clear identification markers (references to experts or institutions, or associations with Nazism or communism), annotators differed in marking span boundaries. Lower agreement levels appeared in “doubt,” “uncertainty” (or “intentional vagueness”), “emotional expression,” and “simplification.” Despite being individually straightforward to identify, annotators frequently disagreed, due to overlapping characteristics, leading to annotations marked as overlapping. “Uncertainty”, involving vague language, similarly presents identification challenges akin to “whataboutism/red herring/straw man.”

**Table 5:** Gamma (γ) inter-annotator agreement between annotators for each technique spotting spans alone (spans) and spotting spans + labelling (+labels).

Technique	Spans (γ_s_)	+labels (γ_sl_)
Emotional Expression	0.23	0.23
Whataboutism/Red Herring/Straw Man	0.13	0.14
Simplification	0.21	0.21
Intentional Vagueness (Obfuscation)	0.23	0.21
Appeal To Authority	0.37	0.37
Flag-Waving	0.43	0.42
Bandwagon	0.43	0.38
Doubt/Smears	0.30	0.30
Reduction ad Hitlerum/Stalinum	0.37	0.39
Repetition	0.50	0.49

### Narratives labelling validation

We have also evaluated a match between annotators $\{{a}_{1},{a}_{2},{a}_{3},{a}_{4},{a}_{5}\}$ narratives for each article using the Jaccard similarity metric. The average values of the metric and its standard deviations are presented in Table 6. The results indicate that average pairwise Jaccard similarity scores ranged from 0.30 to 0.57, suggesting a generally moderate level of agreement. However, all annotator pairs exhibited substantial variability across articles, with standard deviations consistently exceeding 0.42. This high variability can be attributed to the multi-label nature of the task, where annotators were permitted to assign multiple narratives to each article. Instances in which one annotator labeled a narrative that another did not initially identify were addressed during the final discussion phase of the cross-annotation methodology. In most cases, annotators reached a consensus to retain multiple narrative labels in the consolidated annotation^52^.

**Table 6:** Average values and standard deviations of the Jaccard similarity metric between annotators based on narrative labelling.

	*a*_1_	*a*_2_	*a*_3_	*a*_4_	*a*_5_
${{\boldsymbol{a}}}_{{\boldsymbol{1}}}$	—	0.565 ± 0.46	0.3 ± 0.43	0.566 ± 0.46	0.54 ± 0.43
${{\boldsymbol{a}}}_{{\boldsymbol{2}}}$	0.565 ± 0.46	—	0.377 ± 0.45		
${{\boldsymbol{a}}}_{{\boldsymbol{3}}}$	0.3 ± 0.43	0.377 ± 0.45	—		
${{\boldsymbol{a}}}_{{\boldsymbol{4}}}$	0.566 ± 0.46			—	0.556 ± 0.42
${{\boldsymbol{a}}}_{{\boldsymbol{5}}}$	0.54 ± 0.43			0.556 ± 0.42	—

**Table 7:** HALP-PROP corpus structure.

File	Field name	Description
Annotations.csv (1000 records)	index	An article number
	heading	Media outlet article headline
	content	Media outlet article content, without HTML formatting in the Lithuanian language
	is_propaganda	Label for the content. It could be one of the following values: • “yes” for text marked as propaganda • “no” for text that is not marked as propaganda • “propagandaCitation” for an article that consists only of propaganda citation • “nonDeterminable”/“Unclear” for an article that could not be defined as propaganda or not propaganda
	narratives	Narratives found in the content of the article. A detailed description of each narrative can be found in the section “Selection of Propaganda Narratives”. The content could have a few narratives from the list: 1. “disinformationAboutTheWarInUkraine” 2. “delegitimizationOfTheLithuanianState” 3. “underminingTheLithuanianArmedForces” 4. “erosionOfTrustInLithuanianInstitutions” 5. “attacksOnWesternInstitutionsAndAlliances” 6. “declineOfWesternCivilization” 7. “authoritarianModelPromotion” 8. “U.S.DeclineAndWashingtonHegemony” 9. “geopoliticalReorderingAndTheNewWorldOrder” 10. “weaponizationOfMigrationAndRefugees” 11. “revivalOfLitvinism”
	custom_narratives	It could be a list of other narratives in Lithuanian language identified by the annotators
	techniques	The list of techniques found in the article content. Each item on the list has a structure as follows: • “start” – the beginning of the technique • “end” – the end of the technique • “technique” – abbreviation of the technique found in Table 2 • “text” – the part of article content corresponding to the technique
Primary_filter.csv (2870 records)	index	An article number
	heading	Media outlet article headline
	content	Media outlet article content, without HTML formatting in the Lithuanian language
	is_propaganda	Has the same values as in Annotations.csv

## Data Availability

The corpus is available on National Open Access Research Data Archive (MIDAS) 10.18279/MIDAS.260057.

## Code availability

No custom code was used.
